# Bone mineral density by digital X-ray radiogrammetry is strongly decreased and associated with joint destruction in long-standing Rheumatoid Arthritis: a cross-sectional study

**DOI:** 10.1186/1471-2474-12-242

**Published:** 2011-10-24

**Authors:** Helena Forsblad-d'Elia, Hans Carlsten

**Affiliations:** 1 Sahlgrenska Academy at University of Gothenburg, Department of Rheumatology and Inflammation Research, Centre for Bone and Arthritis Research, Box 480, S-405 30 Gothenburg, Sweden

## Abstract

**Background:**

The aims were to explore bone mineral density (BMD) by digital X-ray radiogrammetry (DXR) in postmenopausal women with long-lasting rheumatoid arthritis (RA) in relation to dual x-ray absorptiometry (DXA)-BMD, joint destruction by conventional radiographs and disease related variables in a cross-sectional study.

**Methods:**

Seventy-five postmenopausal women with RA were examined by DXA measuring DXA-BMD of the forearm, total hip and lumbar spine, by scoring joint destruction on plain radiographs by the method of Larsen and by DXR-BMD in metacarpals two to four. The DXR-BMD results of the RA women were compared with an age and sex-matched reference database. A function of DXR-BMD in relation to age and disease duration was created. Associations were investigated by bivariate and multiple linear regression analyses.

**Results:**

DXR-BMD was strongly decreased in RA patients compared to the reference database (p < 0.001). Calculations showed that DXR-BMD was not markedly influenced the first years after diagnosis of RA, but between approximately 5-10 years of disease there was a steep decline in DXR-BMD which subsequently levelled off. In multiple regression analyses disease duration, CRP and DXR-BMD were independent variables associated with Larsen score (R^2^= 0.64). Larsen score and BMD forearm were independent determinants of DXR-BMD (R^2 ^= 0.79).

**Conclusions:**

DXR-BMD was strongly reduced and associated with both Larsen score and DXA-BMD forearm in these postmenopausal women with RA implying that DXR-BMD is a technique that reflects both the erosive process and bone loss adjacent to affected joints.

## Background

Rheumatoid arthritis (RA) is a common inflammatory rheumatic disease characterized by synovitis and by development of cartilage destruction in the joints, erosions in the subchondral bone at the joint margins and by periarticular osteopenia adjacent to inflamed joints [1,2]. In addition, generalized osteoporosis affecting the axial and appendicular bones is a well-known phenomenon in RA as demonstrated by decreased bone mineral density (BMD) in several studies [3-5]. Also, the risk of fractures in RA is elevated [6-8].

Conventional radiographs are the standard method for detection and quantification of joint destruction in RA [9,10]. However, the disadvantages of conventional imaging are its limited sensitivity in early joint space narrowing and periarticular osteoporosis. Bone loss less than around 30% cannot be detected on plain radiographs [11]. The scoring systems are also time consuming, and suitable in particular for experts.

Radiograph-assessed cortical bone width as a measure of bone strength and bone loss was proposed in 1960 using radiographs of the hand bones [12,13]. Refinement in radiogrammetry increased its precision and the development of automated algorithms for image assessment has led to an increased interest in the technique [14]. In RA, localized bone involvement in the hands can be measured by digital X-ray radiogrammetry (DXR), which gives an estimate of cortical hand BMD in the metacarpals two to four. Quantification of the localized periarticular osteoporosis has been proposed to be an outcome measure in monitoring treatments in early RA [15,16] and to be able to predict future radiographic joint damage [17-19]. Localized bone loss in early RA has been found to be better detected by DXR-BMD than by dual x ray absorptiometry (DXA) - BMD [20]. DXR-BMD may also be used in fracture risk assessment [21].

So far, there is limited knowledge of DXR-BMD in long-standing RA, but interestingly, Haugeberg et al found in 2004 that DXR-BMD was a strong indicator of joint damage and of vertebral and non-vertebral fracture risk in RA women with disease duration of ≥ 5 years [22].

The aims of this cross-sectional study were to explore DXR-BMD in postmenopausal women with established RA in relation to DXA-BMD, radiological joint destruction, measures of disease activity and functional status.

## Methods

### Patients

Eighty-eight postmenopausal women with RA aged between 45-65 years were included in a trial which has been described previously [23]. Patients had an active disease which met at least two of the following criteria: at least 6 painful, at least 3 swollen joints out of 28, erythrocyte sedimentation rate (ESR) >20 mm/h and C-reactive protein (CRP) >10 mg/L. A maximum daily dose of 7.5 mg of prednisolone was accepted. Patients fulfilled the American Rheumatism Association 1987 revised criteria for adult RA [24]. DXR-BMD was assessed in 75 women out of the 88 women who were included in the trial. As a consequence of prostheses, osteosynthetic materials and the position of the hands it was not possible to measure DXR-BMD on all radiographs of the 88 women. Thus, this report presents results of the 75 RA women.

### Assessment of outcome variables

*Disease Activity *was assessed by the Disease Activity Score 28 (DAS 28) [25].

*Physical disability *was evaluated by the Health Assessment Questionnaire (HAQ) where 0 implies no handicap and 3, severe disability [26,27].

*Radiographs *of the hands, wrists and forefeet were obtained. Forty joints in the hands and feet were scored. (in the hands, proximal interphalangeal joints of digits 1-5, metacarpophalangeal joints of digit 1-5, wrist areas 1-4, in the feet the interphalangeal joints of digit 1 and the metatarsophalangeal joints of digit 1-5). Larsen score was evaluated by Dr Arvi Larsen [28]. Each joint was scored from 0 (normal) to 5 (maximal destruction). The scores for every patient were summarised and divided by the number of examined joints to give the mean Larsen score ranging from 0-5. Larsen score obtained from the hands and wrists but not from the forefeet are also given. Erosive disease is defined as a score of at least 2 in at least one joint.

*DXR-BMD *was measured on standard radiographs of the hands by a computer version (dxr-online, Sectra, Linköping, Sweden) [29] of the traditional technique of radiogrammetry [12]. The computer automatically recognises regions of interest around the narrowest part of the second, third and fourth metacarpal bones of the hands. In each region the cortical thickness and bone width are measures 90-204 times per centimeter. The final BMD estimate is defined as : DXR-BMD = c × VPA_mc _× (1-p) where c is a constant (determined such that DXR-BMD on average is equal to the mid-distal forearm region of the Hologic QDR-2000 device (Hologic, Waltham, MA, USA)). VPA is the volume per projected area and p is porosity [29].

The mean values of DXR-BMD from left and right hands were used when both radiographs were available. Sixty-three out of 75 values are mean DXR-BMD, 9 left and 3 right hand values. The value of these 75 measurements is given when not indicated otherwise.

The results of DXR-BMD in left hand in our patients was compared with DXR-BMD measured in the non-dominant hand in a large normative reference database comprising of 822 North American Caucasian Females, 20 - 79 years old [30]. Our findings of DXR-BMD are presented as absolute values (g/cm^2^). Z-score, which is related to the normative reference database, is also given of the left hand.

#### A function of DXR-BMD in relation to age and disease duration

A function of Z-score was formed based on results of the individual patients Z-score, age and disease duration. In order to find out if Z-score was influenced differently depending on diverse intervals of disease duration a piece-wise linear regression with break points at the duration of 5 and 10 years was obtained.

*DXA-BMD *at left total forearm, left total hip and lumbar spine (L1-4) was measured by DXA, Hologic QDR-4500A (Hologic Inc., Bedford, MA, USA). The in-vivo precisions were found to be 0,4% both at the lumbar spine and total hip and according to the manufacturer the in-vivo precision of DXA of the forearm was <1%. It was not possible to measure all skeletal sites in every patient due to presence of prostheses and osteosynthetic materials. The results are presented as absolute values (g/cm^2^) and Z-scores, related to sex and age matched references.

#### Biochemical analyses of blood samples

Venous blood samples were drawn in the morning after an overnight fast and stored at -70°C until time of analysis.

*Routine laboratory tests*: erythrocyte sedimentation rate (ESR), C-reactive protein (CRP), Haemoglobin (Hb) and rheumatoid factor (RF) test were measured using standard laboratory techniques.

#### Carboxyterminal telopeptide fragments of type I collagen

Serum levels of bone resorption derived collagen type I fragments (CTX-I) was measured by a one step ELISA (Nordic Bioscience A/S, Herlev, Denmark) [31]. The detection limit was 0.01 ng/mL. Intra- and inter-assay coefficient of variations (CV) of the serum CTX-I assay was 5.4 and 6.2% respectively.

#### Carboxyterminal telopeptide of type I collagen

Radioimmunoassay (RIA) was used for the quantitative determination in serum of the bone resorption marker ICTP (Orion Diagnostica, Espoo, Finland). The detection limit of the test was 0.5 ng/mL and the intra- and inter-assay CV were <8% according to the manufacturer and <6% at our laboratory.

### Ethical aspects

All patients gave informed written consent according to the Declaration of Helsinki. The study was approved by the Regional Ethics Committee in Gothenburg, R528-97.

### Statistics

Analyses were performed using SPSS version 16.0 (SPSS Inc., Chicago, IL). Descriptive statistics are presented as mean and standard deviations (SD). Bivariate correlations were assessed by Pearson's Correlations (r) and by Spearman's Correlation (r_s_) when appropriate. Multiple linear regression analyses were used by a stepwise (forward) method to explore the relationships between DXR-BMD, DXA-BMD forearm, Larsen score and Larsen score (hand) as dependent variables and the demographic and disease related variables listed in Table 1, which had shown significant correlations in the simple regressions. All tests were two tailed and p < 0.05 was considered statistically significant.

**Table 1:** Characteristics of the patients

	n	Characteristics
Age, years	75	57.6 ± 5.2
Height, cm	75	163.2 ± 6.4
Weight, kg	75	67.2 ± 12.7
Disease duration, years	75	14 (7, 20)
Disease modifying anti rheumatic drugs ( n, %)	75	64, 85%
Glucocorticosteroid treatment ( n, %)	75	18, 24%
Health Assessment Questionnaire, score	75	1.00 (0.38, 1.50)
Disease Activity Score 28, score	74	5.3 ± 1.0
Haemoglobin,(g/L	75	129 ± 12
Erythrocyte sedimentation rate, mm/h	74	28 ± 17
C-reactive protein, mg/L	74	17 ± 17
Mean Larsen score, score	72	1.21 ± 0.96
Mean Larsen score, hands, score	72	1.18 ± 1.02
DXA-BMD, forearm, g/cm^2^	70	0.47 ± 0.10
DXA-BMD, forearm, Z-score	70	-0.59 ± 1.94
DXA-BMD, total hip, g/cm^2^	74	0.78 ± 0.15
DXA-BMD, total hip, Z-score	74	-0.69 ± 1.21
DXA-BMD, lumbar spine, g/cm^2^	74	0.86 ± 0.13
DXA-BMD, lumbar spine, Z-score	74	-0.44 ± 1.14
DXR-BMD, left hand, g/cm^2^	72	0.45 ± 0.08
DXR-BMD, right hand, g/cm^2^	66	0.46 ± 0.09
DXR-BMD, mean values, g/cm^2^	63	0.46 ± 0.08
DXR-BMD, mean, left or right hand, g/cm^2^	75	0.45 ± 0.09
CTX-I, ng/mL	68	0.61 ± 0.36
ICTP, ng/mL	66	4.9 ± 1.9

Demographic variables, clinical and laboratory measurements of disease activity, radiographic score (Larsen), bone mineral density measurements by dual energy x-ray absorptiometry and by digital x-ray radiogrammetry in postmenopasusal women with RA.

Values are means ± SD or median (IQR) when not stated otherwise, n = number of patients with available data.

CTX-I = C-terminal telopeptide fragments of type I collagen, ICTP = C-terminal telopeptide of type I collagen

## Results

### Patients

In table 1, characteristics of the patients are displayed. All women were postmenopausal. The mean age at menopause was 49.1 ± 3.5 years and the mean time since menopause was 8.4 ± 5.7 years. Disease modifying anti rheumatic drugs (DMARD) were used by 85%. Methorexate was the most common DMARD used by 28 (37%) women. Low-dose prednisolone was used by 18 (24%) patients, 1.25. mg to 7.5 mg/day. Sixty-three out of 75 (88%) had an erosive disease and 61 (81%) had positive tests for RF.

### DXR-BMD in RA women in comparison with reference population

DXR-BMD was compared with a normative reference database [30]. The Z-score, non dominant hand, of the female general population has the mean 0 and the standard deviation 1. The mean Z score of the RA women, left hand, was -2.27 ± 1.69 SD, much lower than zero of the DXR manufactures reference population (p < 0.001).

### DXR-BMD in relation to age and disease duration

A function of Z-score was formed based on results of the individual patients Z-score, age and disease duration.

Z-score = -0.315 - 0.0155⋅age - 0.0702⋅duration (multiple correlation coefficient 0.49).

The function demonstrates that disease duration has a much larger and significant impact on Z-score as compared to age of the patients. Therefore, the independent variable age was excluded and a linear regression comprising disease duration as the only independent variable was formed.

Z-score = -1.193 - 0.0712⋅duration (correlation coefficient -0.49, p < 0.001).

In addition, in order to find out if Z-score was influenced differently depending on diverse intervals of disease duration a piece-wise linear regression with break points at the duration of 5 and 10 years was obtained.

Z-score = -0.6796 + 0.0028⋅min(duration, 5) - 0.4177⋅max(min(duration-5, 10-5), 0) -0.0209⋅max(duration-10, 0) (multiple correlation coefficient 0.60).

**Figure 1:**
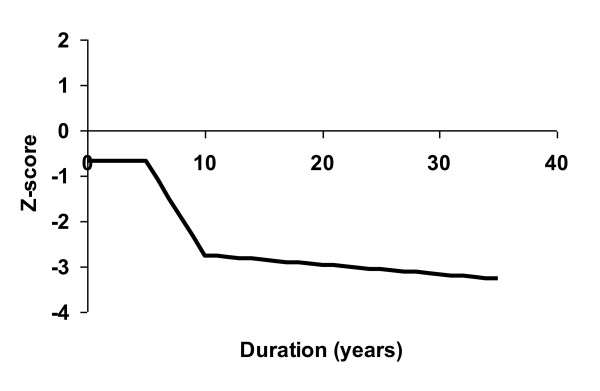
**DXR-BMD in relation to disease duration**. The mean Z-score (SD) of the patients was estimated as piece-wise linear and continuous function of the disease duration. The corresponding function for the general population coincides with the x-axes. (Z-score = -0.6796 + 0.0028 min(duration, 5) - 0.4177 max(min(duration-5, 10-5), 0) -0.0209 max(duration-10, 0) (multiple correlation coefficient 0.60)).

The correlation coefficient in the last function is higher compared with the previous coefficients, indicating a better explanation for the Z-score in the last model. The function shows that during the first years after diagnosis of RA the Z-score is not markedly influenced, but between approximately 5-10 years of disease there is a steep decline in DXR-BMD which subsequently levels off (Figure 1).

From the last relationship we found that the mean Z-score at diagnosis was -0.68. Furthermore, we calculated that the probability to have a Z-score larger than 0 at diagnosis was 31%. Thus, at diagnosis the DXR-BMD did not differ so much from that of the general population.

### Associations between DXR-BMD, DXA-BMD, Larsen score and markers of inflammation and bone turnover

DXR-BMD in left and right hand correlated strongly (r = 0.93). Both DXR-BMD in left hand and in right hand correlated strongly with mean value of left and right hand DXR-BMD (r = 0.98). The mean DXR value in the right hand was 0.46 g/cm^2 ^and in the left 0.45 g/cm^2^, p = 0.053.

**Figure 2:**
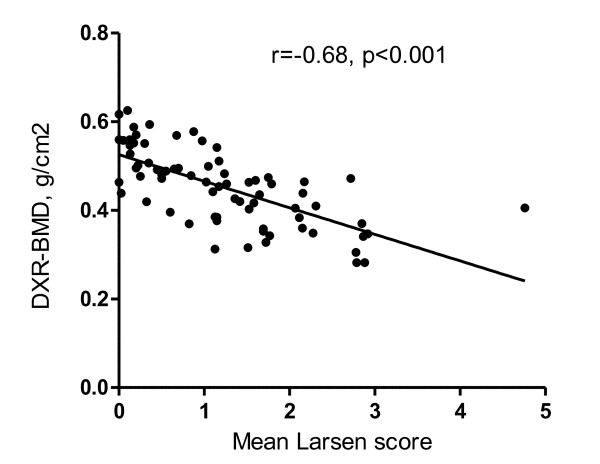
**Relation between joint destruction and DXR-BMD**. Scatter diagrams of mean Larsen score and bone mineral density by digital X-ray radiogrammetry. The regression line is inserted and Pearson's Correlation (r) is given.

Associations between DXR-BMD, DXA-BMD, Larsen score and markers of inflammation and bone turnover are shown in table 2. DXR- BMD was significantly strongly inversely correlated to Larsen score which is illustrated in Figure 2.

**Table 2:** Correlations. Correlations between bone mineral density by digital X-ray radiogrammetry and by dual energy x-ray absorptiometry, total Larsen score, Larsen score of the hands, markers of inflammation and bone turnover in postmenopausal women with rheumatoid arthritis

	Age	Height	Weight	Disease duration	HAQ	DAS 28	ESR	CRP	Hb	CTX-I	ICTP	DXR-BMD	Larsen score	Larsen score hand	DXA-BMD forearm	DXA-BMD total hip	DXA-BMD lumbar spine
DXR-BMD	-0.33** (75)	0.31** (75)	0.38** (75)	-0.45*** (75)	-0.29* (75)	-0.20 (74)	-0.32** (74)	-0.21 (74)	0.31** (75)	-0.22 (68)	-0.28* (66)		-0.68*** (72)	-0.68*** (72)	0.82*** (70)	0.65*** (74)	0.52*** (74)
Larsen score	0.002 (72)	-0.13 (72)	-0.20 (72)	0.46*** (72)	0.42*** (72)	0.33** (71)	0.55*** (71)	0.49*** (71)	-0.39*** (72)	0.29* (66)	0.61*** (63)	-0.68*** (72)		0.97*** (72)	-0.48*** (67)	-0.43*** (71)	-0.17 (71)
Larsen score, hand	0.022 (72)	-0.14 (72)	-0.14 (72)	0.48*** (72)	0.46*** (72)	0.33** (71)	0.54*** (71)	0.48*** (71)	-0.36*** (72)	0.32** (66)	0.65*** (63)	-0.68*** (72)	0.97*** (72)		-0.50*** (67)	-0.42*** (71)	-0.20 (71)
DXA-BMD forearm	-0.32** (70)	0.35** (70)	0.46*** (70)	-0.34** (70)	-0.16 (70)	-0.040 (69)	-0.082 (69)	-0.07 (69)	0.16 (70)	-0.16 (63)	-0.23 (62)	0.82*** (70)	-0.48*** (67)	-0.50*** (67)		0.76*** (69)	0.69*** (69)

Number of patients with available data is given within (). *p < 0,05, **p < 0,01, ***p < 0,001

CTX-I = C-terminal telopeptide fragments of type I collagen, ICTP = C-terminal telopeptide of type I collagen

### Multiple regression analyses

Results of the multiple regression analysis with DXR-BMD, Larsen score, Larsen score hand and DXA-BMD forearm are shown in Table 3. In the analysis with DXR-BMD as the dependent variable Larsen score and BMD forearm were found to be significant independent determinants (R^2 ^= 0.79). If only the BMD measure sites, lumbar spine and hip, that are used in clinical practice evaluating the prevalence of osteopenia/osteoporosis were put into the model, we found that Larsen score (hand), BMD lumbar spine, age and weight were significant independent variables connected with DXR-BMD (R^2 ^= 0.66). Finally, BMD total hip, BMD lumbar spine and DXR-BMD were independent variables connected with, DXA-BMD forearm (R^2^= 0.76). If DXR-BMD was excluded from the analyse BMD total hip and lumbar spine, Larsen score (hand) and age were significant independent variables connected with DXA-BMD forearm (R^2 ^= 0.70)

**Table 3:** Multiple regression analyses

	DXR-BMD			Larsen score			Larsen score hand			DXA-BMD forearm		
**Constant**	**0.28**			**2.74**			**2.22**			**-0.12**		

	**Beta**	**SE**	**P**	**Beta**	**SE**	**P**	**Beta**	**SE**	**P**	**Beta**	**SE**	**P**

Disease duration (years)				0.020	0.007	0.005	0.022	0.007	0.003			
CRP (mg/L)				0.017	0.004	<0.001	0.014	0.005	0.010			
ICTP (ng/mL)							0.092	0.049	0.064			
DXR-BMD (g/cm^2^)				-4.81	0.96	<0.001	-4.74	0.99	<0.001	0.62	0.097	<0.001
Larsen score (score)	-0.049	0.007	<0.001									
BMD forearm (g/cm^2^)	0.47	0.057	<0.001									
BMD total hip (g/cm^2^)										0.15	0.072	0.039
BMD lumbar spine (g/cm^2^)										0.23	0.071	0.002
R^2^	0.79			0.67			0.70			0.76		

Multiple regression analyses of bone mineral density by digital x-ray radiogrammetry and by dual energy x-ray absorptiometry and of joint destruction assessed by Larsen score in postmenopausal women with rheumatoid arthritis.

Beta values are unstandardized coefficients. The R^2 ^is the total variance explained in the model. SE = standard error

ICTP = C-terminal telopeptide of type I collagen

## Discussion

In this group of postmenopausal women with long-term RA, we found, for the first time, that DXR-BMD was significantly and markedly reduced, Z-score -2.27 ± 1.69SD compared to the reference population. The DXR-BMD Z-score was numerically notably lower compared to the DXA-BMD Z-scores in our trial showing Z-score of -0.59 ± 1.94SD in forearm, -0.69 ± 1.21SD in total hip and -0.44 ± 1.14SD in lumbar spine. The finding indicates that the cortical BMD in the metacarpal bones is more influenced by RA as compared to BMD assessed by DXA which measures both cortical and trabecular BMD. The DXA-BMD findings are in line with previous results of BMD assessed by DXA in RA displaying reduced DXA-BMD [4,5,32].

Furthermore, our estimations showed that DXR-BMD was marginally reduced at start of the RA disease and the largest decrease was approximate during 5-10 years after RA debuted. Previous investigations have found decrease in DXR-BMD the first years after RA diagnose [17,18,20,33] and Böttcher et al, who followed RA patients during 6 years, showed a continuous decrease in DXR-BMD [34]. Hoff et al found a reduction of DXR-BMD during 2-years follow-up in both patients with disease duration < 3 years and > 3 years [35]. Jensen et al found, in a study investigating DXR-BMD between groups of RA women stratified according to disease duration, significantly lower DXR-BMD in RA women with disease duration ≥ 10 years compared with patients with disease duration < 2years but not with patients with disease duration between 2 and 10 years [36]. In our study, exclusively women were included. The mean age was 58 years, the mean disease duration was 15 years and the mean age at menopause was 49 years in our trial. Thus, the mean age when RA debuted was 43 years in this group of RA women. The largest fall in DXR-BMD was between 5 and 10 years after the disease started, accordingly between the ages of 48 and 53 years, coinciding with the years surrounding menopause. During this period the production of estradiol diminishes which is associated with accelerated bone loss [37]. The fast decrease in DXR-BMD was based on Z-scores, excluding the influence of menopause as the only cause, although, it could be hypothesised that RA women are more sensitive to menopause and its hormonal alteration concerning influence on disease process and BMD compared to the general population. In accordance, treatment with HRT has been found to increase BMD, retard joint destruction as well as reduce disease activity [23,38,39]. In addition, we recently found that treatment with HRT stabilised DXR-BMD in postmenopausal RA [40]. Interestingly, also the peak incidence of RA onset corresponds with the years surrounding the menopause when serum levels of estradiol fall [41,42].

There was a tendency of significantly higher DXR- BMD values in the right hand as compared with the left hand although the correlation between right and left hand DXR-BMD values was very high (0.93) in accordance with others [36].

DXR-BMD was strongly associated with Larsen score evaluated on hands and feet and on hands only. DXR-BMD was also strongly correlated to BMD measured by DXA, in particular in the forearm, also found by Black et al who obtained correlations between DXR-BMD and DXA-BMD at the wrist of 0.90 and of 0.61 at the hip [30].

In multiple regression analyses Larsen score and BMD forearm remained the most important independent determinants of DXR-BMD displaying a high R^2 ^value whereas DXR-BMD, ESR and disease duration were associated with Larsen score. Based on these results, DXR-BMD seems to be a technique that reflects radiographic joint destruction powerfully and at the same time, bone loss both in the axial and appendicular skeleton, in agreement with others [36,43].

To assess radiographic joint destruction by traditional validated scoring system is time consuming and suitable in particular for experts. In contrast DXR-BMD is an objective and convenient digitalized method quantifying localized bone-loss. Thus, DXR-BMD has been proposed to be an outcome measure in monitoring treatments in early RA [15,16] and have been found to be able to predict future radiographic joint damage [17-19]. The role of DXR-BMD in predicting progressive joint destruction in established RA remains to be explored. Recently, Book et al found that low DXR-BMD in RA patients predicted overall mortality, which supports DXR-BMD as a promising prognostic tool [44]. Also, it remains to be elucidated in long-lasting RA, if the technique is suitable to be used as a disease outcome measure in clinical trials and in ordinary clinical practice evaluating the effects of DMARDs on the peripheral skeleton. Thus, further prospective longitudinal studies of the role of DXR-BMD in established RA are needed. Another intriguing question is if drugs that target different biological mechanisms affect DXR-BMD diversely or not.

## Conclusions

We have found, in these postmenopausal women with RA that DXR-BMD was markedly decreased. In multiple regression analyses, Larsen score was strongly associated with DXR-BMD and DXR-BMD was strongly connected with both Larsen score and DXA-BMD implying that DXR-BMD is a method that in particular reflects the erosive process in addition to bone loss adjacent to affected joints.

## List of Abbreviations

BMD: bone mineral density; CRP: C-reactive protein; ICTP: C-terminal telopeptide of type I collagen; CTX-I: C-terminal telopeptide fragments of type I collagen; DAS 28: disease activity score 28; DMARD: disease modifying anti rheumatic drug; DXA: dual x-ray absorptiometry; DXR: digital X-ray radiogrammetry; E_2_: estradiol; ESR: erythrocyte sedimentation rate; Hb: Haemoglobin; HRT: hormone replacement therapy; RA: rheumatoid arthritis; RF: rheumatoid factor

## Competing interests

The authors declare that they have no competing interests.

## Authors' contributions

HF-d'E conceived the study, participated in its design and coordination, performed most of the statistical analyses and drafted the manuscript. HC participated in study design, interpretation of data and revision of the manuscript. All authors read and approved the final manuscript.

## Pre-publication history

The pre-publication history for this paper can be accessed here:

http://www.biomedcentral.com/1471-2474/12/242/prepub
